# A practical approach to procedural planning for robot-assisted segmentectomy using a lobectomy-based surgical simulator

**DOI:** 10.1007/s11748-026-02343-2

**Published:** 2026-06-25

**Authors:** Keiji Yamanashi, Ryo Miyata, Takeshi Kawaguchi, Masatsugu Hamaji

**Affiliations:** Department of Thoracic and Cardiovascular Surgery, Nara Medical University School of Medicine, 840 Shijo-cho, Kashihara, Nara 634-8522 Japan

**Keywords:** da Vinci surgical simulator, Robot-assisted thoracic surgery, Segmentectomy

## Abstract

Preoperative planning for robot-assisted segmentectomy requires accurate understanding of segmental anatomy and the sequence of vascular and bronchial division. Although three-dimensional computed tomography provides detailed anatomical information, it does not allow stepwise prediction of intraoperative anatomical changes. We applied a lobectomy-based da Vinci surgical simulator for preoperative planning of robot-assisted apical–anterior segmentectomy. A 62-year-old woman with a 10-mm pulmonary nodule suspected to be stage IA1 non-small cell lung cancer underwent the procedure. The method focuses on rehearsing the sequence of dissection and anticipating post-division anatomical relationships rather than replicating patient-specific anatomy. Although the procedure can be performed without simulation, this approach may provide additional value by enabling procedural rehearsal and reducing intraoperative uncertainty. The simulated workflow closely corresponded to intraoperative findings, suggesting that this approach may serve as a practical adjunct to conventional imaging in selected cases.

**Supplementary Information:**

The online version contains supplementary material available at https://doi.org/10.1007/s11748-026-02343-2.

## Introduction

Robot-assisted surgery has become increasingly common, and surgical simulations are widely used for training and preoperative planning, allowing surgeons to rehearse procedures and refine technical skills [1]. Simulators for robot-assisted lobectomy have been developed and used for preoperative rehearsal in thoracic surgery. The SimNow platform (Intuitive Surgical, Sunnyvale, CA) is a robotic surgical simulator integrated with the da Vinci system and equipped with procedure-specific modules, including a lobectomy module [2]. It reproduces surgeon console operation in a real-time virtual environment, enabling independent practice of robotic surgical skills and procedural workflows in a non-clinical setting.

In the treatment of lung cancer, anatomical segmentectomy for small peripheral tumors has gained popularity following the results of the JCOG0802 trial [3]. However, data from randomized trials such as the CALGB 140,503 suggest that wedge resection is still more commonly performed than segmentectomy for these tumors [4], indicating that opportunities for structured training in segmentectomy may remain limited in clinical practice. One possible reason is the technical complexity of segmentectomy, particularly for atypical segments. Simulation-based approaches may help overcome these limitations by enhancing surgical understanding and facilitating procedural planning.

In this report, we describe a practical approach to preoperative procedural planning for robot-assisted atypical segmentectomy using a lobectomy-based surgical simulator. This method emphasizes rehearsal of the sequence of dissection and anticipation of post-division anatomy, rather than replication of patient-specific structures.

## Technique

### Patient

**Fig. 1:**
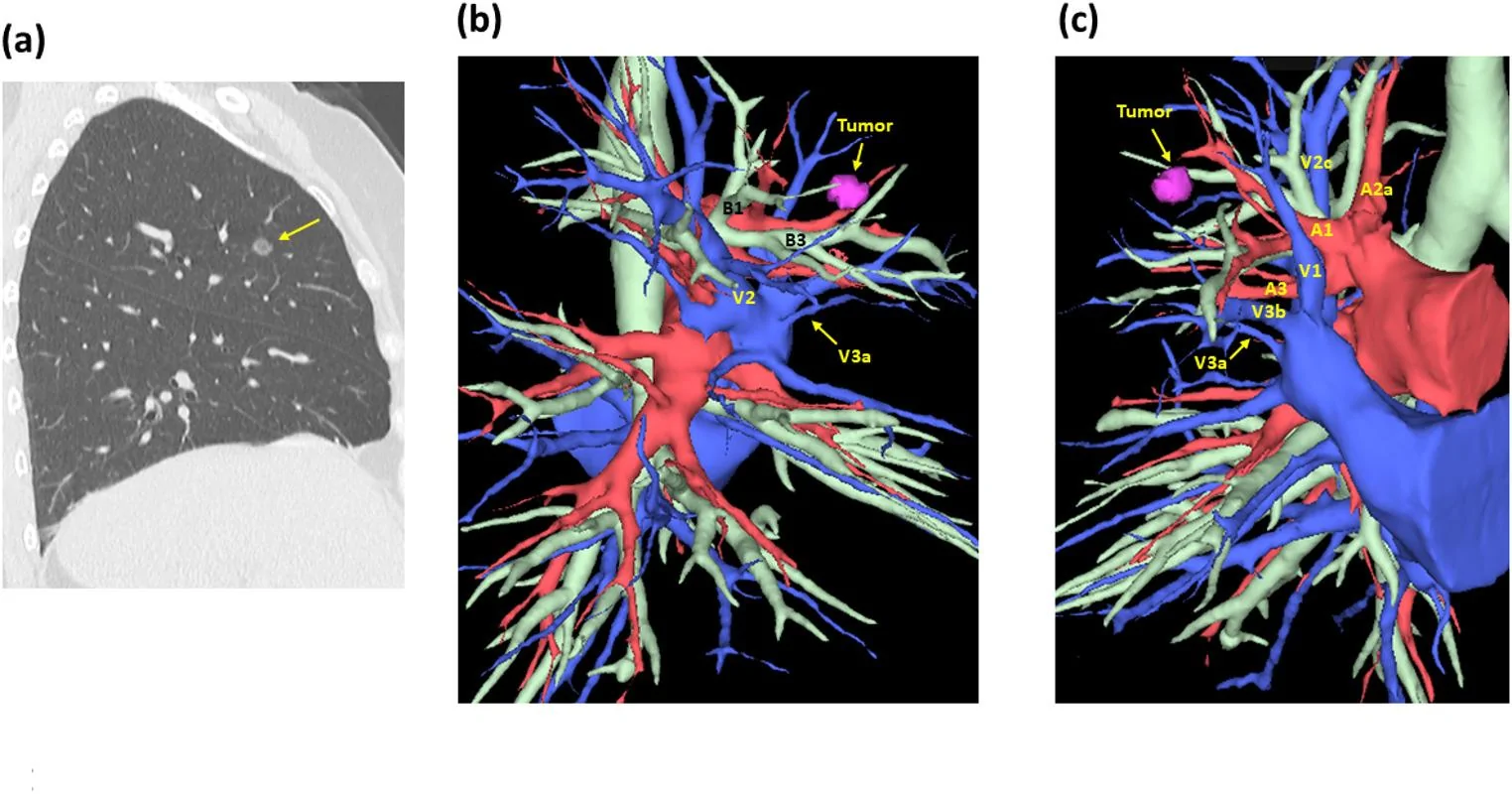
Chest computed tomography demonstrates a pulmonary nodule (arrow) between the right apical and anterior segments (**a**). Three-dimensional computed tomography demonstrates the pulmonary vessels and bronchi (**b**,** c**)

A 62-year-old woman was referred to our department after a contrast-enhanced chest computed tomography (CT) scan incidentally revealed a 10-mm part-solid nodule (consolidation-to-tumor ratio ≤ 0.25) in the right upper lobe (Fig. 1a). The lesion was suspected to be stage IA1 non-small cell lung cancer. Therefore, an apical–anterior segmentectomy was planned based on the location of the nodule and the anatomical variations of the segmental pulmonary arteries, pulmonary veins (PVs), and bronchi (Fig. 1b, c).

### Preoperative simulation using the da Vinci surgical simulator

Although three-dimensional computed tomography (3D-CT) provides valuable anatomical information, apical–anterior segmentectomy is technically demanding and involves atypical segmental anatomy. To better anticipate post-division anatomical relationships and facilitate identification of the intersegmental plane, we performed a preoperative simulation using the da Vinci surgical simulator (Supplemental Video S1). The simulation was performed by the operating surgeon alone and required approximately 40 min to complete. Despite being designed for lobectomy and lacking patient-specific anatomy, the simulated right upper lobe shares key anatomical features with the target segments, allowing rehearsal of vascular and bronchial division, as well as intersegmental plane formation.

**Fig. 2:**
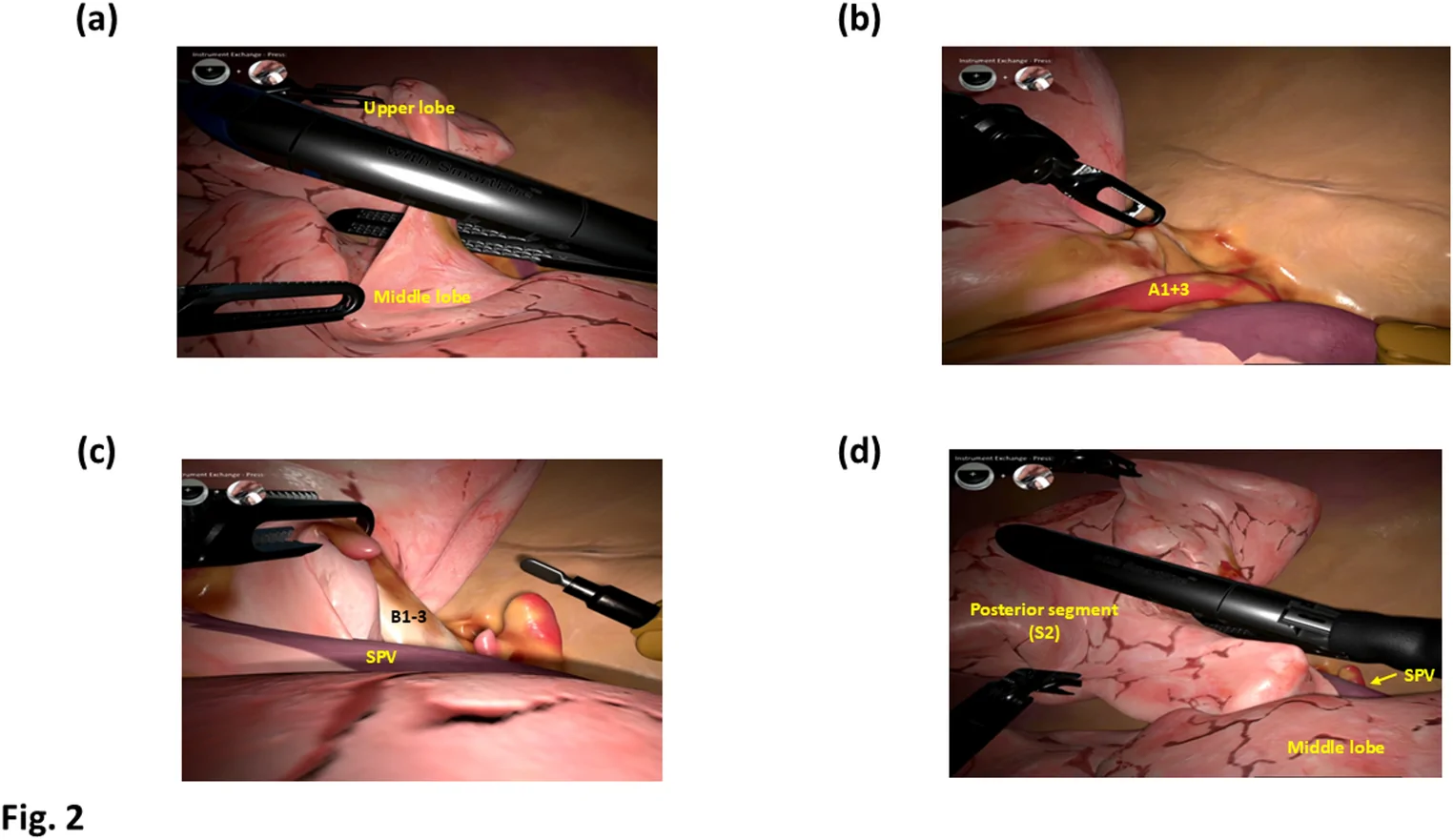
Simulation: Division of the fissure between the upper and middle lobes (**a**). Identification of A1 + 3 (**b**). Identification of the right upper bronchus along the superior pulmonary vein (**c**). Division of the intersegmental plane with robotic staplers along the superior pulmonary vein (**d**)

For clarity, anatomical structures in the simulation are described in relation to their counterparts in the actual procedure. First, the fissure between the upper and middle lobes was divided (Fig. 2a), and the caudal side of the superior PV (V2 in the actual procedure) was exposed. After exposing the cranial side of the superior PV, A1 + 3 (A3 in the actual procedure) was identified (Fig. 2b) and divided, followed by encirclement and division of A2 (A1 in the actual procedure). The superior PV was dissected further distally, allowing identification of the right upper bronchus (B1 + 3 in the actual procedure) (Fig. 2c), which was subsequently divided. Finally, the intersegmental plane was divided by sequential firing of a stapler along the superior PV (Fig. 2d).

### Surgical procedure

**Fig. 3:**
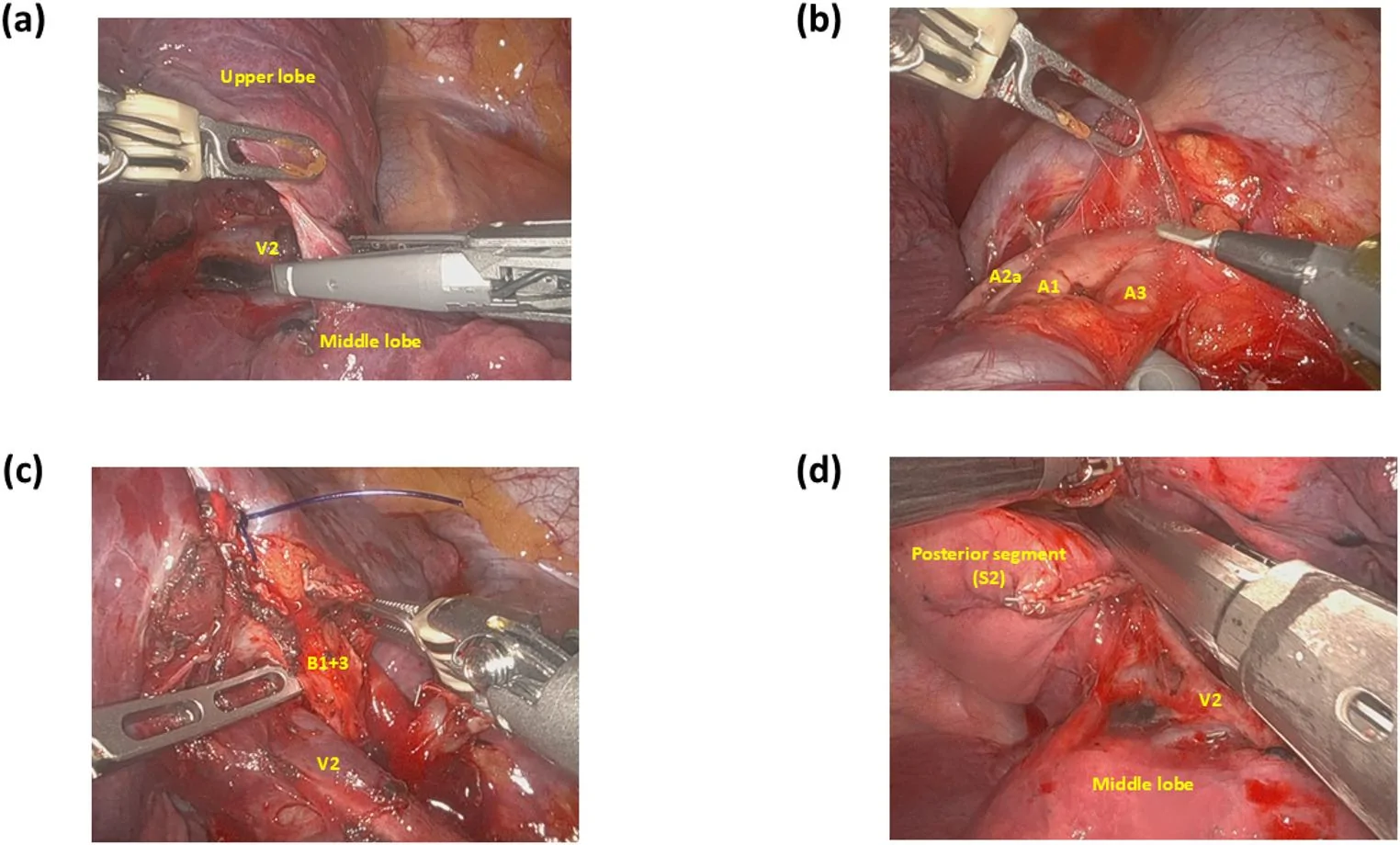
Actual procedure: Dissection of the fissure between the upper and middle lobes (**a**). Identification of A3, A1, and A2a (**b**). Identification of B1 + 3 along V2 (**c**). Division of the intersegmental plane with staplers along V2 (**d**)

A 4-cm incision was made in the sixth intercostal space and three additional ports were placed in the eighth intercostal space. The operation began with dissection of the fissure between the upper and middle lobes. V3a was identified and divided, and the fissure was dissected along V2 (Fig. 3a). After exposing the cranial border of the superior PV, V1 + 3b was divided. The upper lobe was then retracted caudally, revealing A3, A1, and A2a caudal to the azygos vein (Fig. 3b). A3 was isolated and divided. While preserving A2a, A1 was isolated and divided. V2 was dissected further distally, allowing identification and isolation of B1 + 3 (Fig. 3c), which was subsequently divided.

Indocyanine green (10 mg) was administered intravenously, and the intersegmental plane was identified using the Firefly mode and divided with staplers (Signia™ linear stapler, Medtronic, Minneapolis, MN) along V2 (Fig. 3d). Subsequently, the specimen was retrieved. The intraoperative findings were consistent with the simulated procedural workflow. The total operative time was 2 h and 38 min (Supplemental Video S2), with an estimated blood loss of less than 5 mL.

Histopathological examination revealed minimally invasive adenocarcinoma without lymph node metastasis. The chest tube was removed on postoperative day 1, and the patient was discharged on postoperative day 4. A chest X-ray obtained one month postoperatively showed adequate expansion of the right lung (Supplemental Figure S1).

## Discussion

This report describes a practical use of a lobectomy-based surgical simulator for procedural planning in robot-assisted atypical segmentectomy. The main limitation of this approach is the lack of patient-specific anatomy, which has been considered a barrier to its use in preoperative planning. However, this case highlights a clinically relevant role of simulation: supporting procedural anticipation rather than anatomical replication.

 From a practical perspective, the annual cost of the SimNow system is approximately USD 13,000 and includes the simulator hardware together with access to all available simulation training modules [2], including the lobectomy module used in this study. Once the system is available, the simulation can be initiated within approximately 2 min without additional personnel.

In segmentectomy, predicting changes in anatomical relationships after sequential division of segmental vessels and bronchi remains challenging. Although 3D-CT provides detailed anatomical information [5, 6], it does not allow stepwise prediction of these changes, which may lead to uncertainty in the order of dissection and identification of the intersegmental plane.

This approach addresses this limitation by focusing on procedural rehearsal. The sequence of dissection can be rehearsed and the resulting anatomy can be anticipated. In our experience, the simulated workflow closely corresponded to intraoperative findings, and the procedure was completed without modification of the planned sequence. Although the procedure can be performed without simulation [7, 8], this approach may add value by enabling rehearsal and reducing intraoperative uncertainty.

 Because this was the first robot-assisted apical–anterior segmentectomy performed at our institution, comparison with similar institutional cases was not possible. Recent studies of robotic complex segmentectomy reported a median operative time of 220 min with a median blood loss of 10 mL [7], while another study reported a mean operative time of 174 min [8]. In the present case, the operative time was 158 min with an estimated blood loss of less than 5 mL. Although direct comparisons are limited by the single-case nature of this report, the operative outcomes appeared slightly favorable compared with those reported in the recent literature.

Although patient-specific simulation would be ideal, it is not yet widely available [5–8], and the lobectomy-based simulator does not fully reproduce segmentectomy-specific anatomy. In this case, the simulated procedure was not an exact segmentectomy; however, it provided a close anatomical approximation of the planned apical–anterior segmentectomy. Further development of surgical simulators, particularly those incorporating more detailed segmental anatomy of pulmonary vessels and bronchi, may improve their accuracy and clinical applicability in thoracic surgery.

In summary, a lobectomy-based surgical simulator may support procedural planning of segmentectomy by enabling rehearsal of dissection and anticipation of intraoperative anatomical changes. The simulated workflow corresponded to intraoperative findings, suggesting a practical adjunct role to conventional imaging in selected cases.

## Supplementary Information

Below is the link to the electronic supplementary material.

Supplementary Material 1

Supplementary Material 2

Supplementary Material 3
